# Klinische und echographische Untersuchungsbefunde bei Patienten mit Carotis-Sinus-cavernosus-Fisteln

**DOI:** 10.1007/s00347-020-01310-3

**Published:** 2021-01-18

**Authors:** L. Hübner, T. Struffert, C. Y. Mardin, T. Engelhorn, L. Holbach, J. Weller, B. Hohberger, G. Gusek-Schneider

**Affiliations:** 1 Universitätsaugenklinik Erlangen, FAU Erlangen-Nürnberg, Erlangen, Deutschland; 2 Abteilung für Neuroradiologie, Justus-Liebig-Universität Gießen, Gießen, Deutschland; 3 Abteilung Neuroradiologie, FAU Erlangen-Nürnberg, Erlangen, Deutschland

**Keywords:** Echographie, Rotes Auge, Exophthalmus, Doppelbilder, Vena ophthalmica superior, Echography, Red eye, Exophthalmos, Diplopia, Superior ophthalmic vein

## Abstract

**Hintergrund:**

Die Symptomatik und klinischen Befunde bei Patienten mit Carotis-Sinus-cavernosus-Fisteln sind spezifisch, können aber sehr mild ausgeprägt sein. Wir wollen das diagnostische Potenzial der Echographie der Orbita näher beleuchten.

**Methoden:**

In die retrospektive Studie wurden 25 Patienten mit angiographisch gesicherten Fisteln eingeschlossen. Symptome, klinische Untersuchungsergebnisse und die Darstellbarkeit der V. ophthalmica superior in der orbitalen Echographie wurden gegenübergestellt.

**Ergebnisse:**

Die häufigsten Befunde waren Hirnnervenparesen, episklerale Venenstauung oder Exophthalmus. Wurde eine orbitale Ultraschalluntersuchung durchgeführt, konnte eine erweiterte V. ophthalmica superior zu 100 % nachgewiesen werden.

**Schlussfolgerung:**

Die schnell durchführbare Ultraschalluntersuchung liefert wertvolle Informationen zur Differenzialdiagnose eines therapieresistenten roten Auges. In Kombination mit den 3 Kardinalsymptomen episklerale Venenstauung, Exophthalmus und Hirnnervenparese lässt sich die Diagnose einer Carotis-Sinus-cavernosus-Fistel durch die Echographie untermauern. Die weiterführende Bildgebung kann schließlich zielgerichtet veranlasst werden.

## Abstract (en)

**Background:**

Symptoms and clinical findings in patients with carotid-cavernous fistulas are specific. Nevertheless, they can be very mildly expressed. This study aims to point out the potential diagnostic value of ultrasound of the orbit.

**Methods:**

A total of 25 patients with a reliable angiographic diagnosis of a fistula were reviewed retrospectively. We analyzed the symptoms, clinical findings and demonstrability in ultrasound of the orbit.

**Results:**

The most common clinical findings were nerve palsy, dilation of episcleral vessels and exophthalmos. If an ultrasound had been part of the examination a dilation of the superior ophthalmic vein could be demonstrated in all cases.

**Conclusion:**

The expeditious ultrasound investigation provides valuable information for the diagnosis of red eyes which are resistant to treatment. The examiner has to consider a fistula and perform an ultrasound especially when diplopia has newly occurred. Finally, the expedient neuroimaging can be arranged.

Carotis-Sinus-cavernosus-Fisteln sind pathologische Verbindungen zwischen A. carotis bzw. ihren Ästen und dem Sinus cavernosus. Als Folge der Flussumkehr der in den Sinus cavernosus drainierenden Venen werden in der Literatur als klassische ophthalmologische Symptome und/oder Befunde Exophthalmus, Chemosis, dilatierte Episkleralgefäße und Hirnnervenparesen beschrieben. Verschiedene Differenzialdiagnosen kommen dabei in Betracht. Wir wollen zeigen, dass die Sonographie als eine schnell durchführbare Untersuchung additiv zu den klinischen Befunden wertvolle Informationen liefern kann.

## Hintergrund und Fragestellung

Bei Carotis-Sinus-cavernosus-Fisteln (CCF) handelt es sich um pathologische arteriovenöse Verbindungen zwischen A. carotis bzw. ihren Ästen und Sinus cavernosus. Es resultiert ein Anstieg des Venendrucks im Sinus und der in ihn drainierenden Strukturen. Der Sinus cavernosus erhält Zuflüsse aus V. ophthalmica superior und inferior, aus Sinus sphenoparietalis, V. cerebri medialis superficialis und kleineren kortikalen Venen [1]. Im Sinus cavernosus enthaltene Strukturen sind die A. carotis interna (ACI) mit ihren abgehenden Ästen und der lateral von ihr gelegene N. abducens. N. oculomotorius, N. trochlearis sowie der erste und zweite Ast des N. trigeminus verlaufen etwas geschützter in seiner Wand. Endäste der A. carotis externa bilden Anastomosen mit dem Truncus inferolateralis der ACI [1].

Es werden direkte High-flow- von indirekten Low-flow-Fisteln unterschieden, die gebräuchlichste Klassifikation nach Barrow orientiert sich am arteriellen Zufluss ([2]; vgl. Tab. 1). Während direkte Fisteln häufig posttraumatisch, iatrogen oder nach Ruptur eines Aneurysmas entstehen [3, 4], ist die genaue Ätiologie der indirekten Fisteln ungeklärt. Diskutiert wird ein spontanes Auftreten im Bereich von postthrombotischen oder postinfektiösen Umgehungskreisläufen im venösen Abflussweg [5]. Die klinische Symptomatik hängt im Wesentlichen vom arteriovenösen Shuntvolumen und dem venösen Drainagemuster ab.

**Table 1:** 

Einteilung nach Barrow	Arterielle Zuflüsse
Typ A	Kavernöses Segment der A. carotis interna
Typ B	Durale Äste der A. carotis interna
Typ C	Durale Äste der A. carotis externa
Typ D	Durale Äste der A. carotis interna und externa

Nach anterior in die V. ophthalmica superior drainierende Fisteln verursachen bevorzugt okuläre Symptome, bei Patienten mit nach posterior drainierenden Fisteln stehen neurologische Symptome oder eine Diplopie im Vordergrund [6, 7]. Als typische klinische Zeichen werden eine Proptosis, Injektion der episkleralen Gefäße sowie eine Bindehautchemosis beschrieben. Binokulare Doppelbilder können durch Kompression von Augenmuskelnerven verursacht werden. Ein störendes Ohrengeräusch kann bei venöser Drainage über den Sinus petrosus inferior auftreten [8].

Die digitale Subtraktionsangiographie (DSA) stellt den diagnostischen Standard dar, sie erlaubt die Darstellung der Strömungsverhältnisse und die Planung des therapeutischen Prozedere. Die MR-Angiographie wird in der Regel zuvor als weniger invasive Screeningmethode genutzt (vgl. Übersichtsarbeiten [6, 9]). Additiv kann eine Ultraschalluntersuchung wichtige Informationen geben. Hierbei handelt es sich um eine nichtinvasive und schnell durchführbare Untersuchungstechnik. Bereits im Jahr 1987 beschrieben Keltner et al. [10] die Möglichkeit der Darstellung einer dilatierten V. ophthalmica superior, welche im Gesunden aufgrund des geringen Kalibers häufig nicht auszumachen ist. Besonders im Hinblick auf binokulare Doppelbilder kann diese Untersuchungstechnik zudem genutzt werden, orbitale Tumoren oder eine Augenmuskelverdickung im Sinne einer endokrinen Orbitopathie auszuschließen. Stehen eine (epi)sklerale Injektion und Schmerzen im Vordergrund, kann hier zudem auf Zeichen einer Skleritis posterior wie eine Skleraverdickung oder Flüssigkeitsansammlung im Tenonraum geachtet werden [11].

Seit den Fortschritten in der interventionellen Neuroradiologie hat der endovaskuläre Fistelverschluss die offene chirurgische Technik weitestgehend abgelöst. Die Therapie richtet sich nach der arteriellen Versorgung der Fistel, der venösen Drainage und dem Shuntvolumen (vgl. Übersichtsarbeiten [6, 12]).

In dieser Arbeit soll das Potenzial der orbitalen Echographie in der Diagnostik von Sinus-cavernosus-Fisteln näher beleuchtet werden. Es wurden die Symptome und klinischen Befunde von Patienten mit nachgewiesener Fistel herausgearbeitet. Beurteilt wurde retrospektiv, ob sich bei Patienten mit einer vorliegenden CCF auffällige sonographische Untersuchungsergebnisse zeigten.

## Methoden

In die retrospektive Studie wurden Patienten mit angiographisch gesicherten Carotis-Sinus-cavernosus-Fisteln eingeschlossen, welche von 2001 bis 2016 in unserer Institution untersucht wurden. Dies waren 25 Patienten (18 weiblich, 7 männlich), das mittlere Patientenalter betrug 63 Jahre (22 bis 92 Jahre).

**Figure 1:**
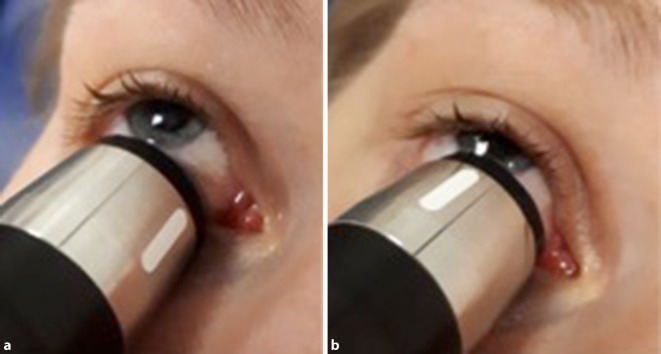

Ausgewertet wurden anamnestische Angaben sowie klinische und apparative Untersuchungsergebnisse. Dabei wurde auf das Vorliegen der in der Literatur beschriebenen typischen Befunde ein besonderes Augenmerk gelegt: Exophthalmus und Injektion der episkleralen Gefäße; alle Patienten wurden zudem orthoptisch auf das Vorliegen von Hirnnervenparesen untersucht. In 14 Fällen (56 %) war eine Echographie der Orbita (Quantel Medical B‑Scan – Cinescan S, 10 MHz B‑Bild, Quantel medical, Cournon-d’Auvergne, France) mit Darstellung der V. ophthalmica superior Bestandteil der ophthalmologischen Untersuchung. Die Vene verläuft zwischen dem M. rectus superior und N. opticus. Um sie darzustellen, wird der Patient gebeten, das Auge nach oben zu rollen. Der Schallkopf wird bei 6 Uhr aufgesetzt, geschallt wird nach superior (Abb. 1). Je nach Ausrichtung des Schallkopfes ist eine transversale oder longitudinale Darstellung möglich. Beurteilt wurde zunächst, ob die Vene darzustellen ist und ob sie dilatiert erscheint. Um das Ergebnis zu quantifizieren, wurde der Durchmesser der Vene im transversalen vertikalen Schnitt gemessen.

## Ergebnisse

### Symptome

Binokulare Doppelbilder waren das am häufigsten vorliegende Symptom bei Erstvorstellung und wurden von 13 Patienten (52 %) beschrieben (Tab. 2). Neun Patienten (36 %) beklagten eine Rötung, jeweils 7 Patienten (28 %) ein hervortretendes Auge und ein Ohrengeräusch. Weitere Symptome waren Visusminderung (24 %), Chemosis (16 %) und Ptosis (16 %). Ein Tinnitus wurde von 7 Patienten (28 %) bemerkt.

**Table 2:** 

Fisteltyp	Exophthalmus	Rötung	Chemosis	Ptosis	Visusabfall	DB	Tinnitus
A (*n* = 6)	2 (33 %)	–	–	1 (17 %)	2 (33 %)	3 (50 %)	3 (50 %)
B (*n* = 2)	–	1 (50 %)	–	–	–	1 (50 %)	–
D (*n* = 17)	5 (29 %)	8 (47 %)	4 (24 %)	3 (18 %)	4 (24 %)	9 (53 %)	4 (24 %)
Gesamt	7 (28 %)	9 (36 %)	4 (16 %)	4 (16 %)	6 (24 %)	13 (52 %)	7 (28 %)

*DB* Doppelbilder

### Klinische Untersuchung

**Figure 2:**
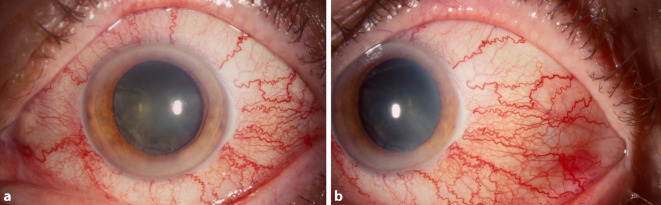

Die häufigsten Befunde in der spaltlampenmikroskopischen Untersuchung waren eine episklerale Venenstauung bei 19 Patienten (76 %) und ein Exophthalmus bei 18 Patienten (72 %) (Tab. 3; Abb. 2). Eine Bindehautchemosis wurde bei 11 Patienten (44 %) beschrieben, eine okuläre Hypertension war bei 10 Patienten (40 %) auffällig. Von den „klassischen“ Befunden (Exophthalmus, episklerale Venenstauung, Hirnnervenparesen) wurden alle Kriterien bei 12 Patienten erfüllt (48 %). Zwei Kriterien wurden bei 10 Patienten erfüllt (40 %), 1 Kriterium bei 3 Patienten (12 %). Im Patientenkollektiv der Echographiegruppe waren im Mittel 2,5 (±0,63) der 3 Kriterien zu beobachten, in der Gruppe ohne Echographie 2,2 (±0,72) der 3 Kriterien.

**Table 3:** 

Fisteltyp	Exophthalmus	BH-Chemosis	Episklerale Venenstauung	OHT	Motilitätsstörungen	aHT
A (*n* = 6)	3 (50 %)	1 (17 %)	3 (50 %)	–	6 (100 %)	2 (33 %)
B (*n* = 2)	2 (100 %)	2 (100 %)	2 (100 %)	2 (100 %)	2 (100 %)	–
D (*n* = 17)	13 (76 %)	8 (47 %)	14 (82 %)	8 (47 %)	14 (82 %)	8 (47 %)
Gesamt	18 (72 %)	11 (44 %)	19 (76 %)	10 (40 %)	22 (88 %)	10 (40 %)

*BH* Bindehaut, *OHT* okuläre Hypertension, *aHT* arterielle Hypertonie

In der Allgemeinanamnese war bei 11 Patienten (44 %) eine arterielle Hypertonie zu finden, selektiv auf Low-flow-Fisteln bezogen waren dies 9 von 19 Patienten (47 %).

Bestandteil der klinischen Diagnostik war stets eine orthoptische Untersuchung. Hirnnervenparesen wurden bei 22 Patienten (88 %) diagnostiziert. Abduzensparesen wurden in 13 Fällen (52 %) beobachtet. Kombinierte Abduzens- und Okulomotoriusparesen wurden bei 4 Patienten (16 %), eine reine Okulomotoriusparese bei 1 Patienten (4 %) und eine nicht näher bezeichnete Ophthalmoplegie in 4 Fällen (16 %) beschrieben, Tab. 4.

**Table 4:** 

Fisteltyp	N.-VI-Parese	N.-III-Parese	N.-VI- und N.-III-Parese	Ophthalmoplegie	Keine Paresen
A (*n* = 6)	2 (33 %)	–	2 (33 %)	2 (33 %)	–
B (*n* = 2)	2 (100 %)	–	–	–	–
D (*n* = 17)	9 (53 %)	1 (6 %)	2 (12 %)	2 (12 %)	3 (18 %)
Gesamt	13 (50 %)	1 (4 %)	4 (15 %)	4 (15 %)	3 (12 %)

### Echographie der Orbita

**Figure 3:**
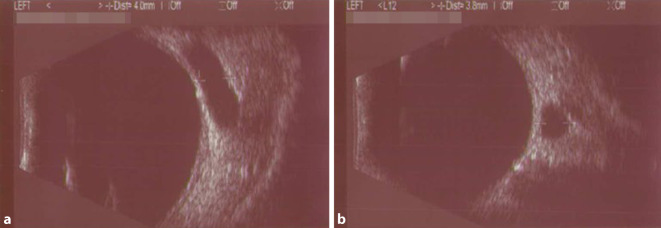

Bei 14 der 25 Patienten (56 %) wurden orbitale Ultraschalluntersuchungen durchgeführt. Die Darstellung der dilatierten V. ophthalmica superior gelang bei allen untersuchten Patienten (100 %), vgl. Abb. 3. Keiner der Untersucher beschrieb einen unauffälligen sonographischen Befund. Die Berechnung des Durchmessers erfolgte in 11 Fällen im transversalen vertikalen Schnitt. Die Werte variierten zwischen 1,0 und 5,1 mm (Mittelwert 3,7 mm ± 1,2 mm), Tab. 5. Die Vene ist dabei über dem N. opticus aufzusuchen. Bei 3 Patienten wurde keine Berechnung durchgeführt, im Untersuchungsbericht wurde jedoch eine „gestaute V. ophthalmica superior“ beschrieben.

**Table 5:** 

Patient	Fisteltyp	Exophthalmus^a^	BH-Chemosis	Episklerale Venenstauung	HN-Paresen	Klinische Befunde (x aus 4)	Ø VOS (mm)
1/m/22	A	Ja (7 mm)	Nein	Ja	Ja	3	5,0
2/w/71	D	Nein (0 mm)	Nein	Ja	Ja	2	1,9
3/w/63	D	Ja (3 mm)	Nein	Nein	Ja	2	4,0
4/m/50	D	Nein (1 mm)	Ja	Ja	Ja	3	1,0
5/w/72	D	Ja (4 mm)	Ja	Ja	Ja	4	4,0
6/w/63	D	Ja (3 mm)	Ja	Ja	Ja	4	„Gestaut“
7/w/24	D	Nein (0,5 mm)	Nein	Ja	Ja	2	4,0
8/m/76	D	Ja (3 mm)	Ja	Ja	Ja	4	3,5
9/w/71	D	Ja (2 mm)	Ja	Ja	Nein	3	„Gestaut“
10/w/72	D	Ja (2 mm)	Ja	Ja	Ja	4	4,5
11/m/61	A	Ja (2 mm)	Nein	Ja	Ja	3	5,1
12/w/60	D	Ja (2 mm)	Nein	Ja	Ja	3	„Gestaut“
13/w/85	B	Ja (2 mm)	Ja	Ja	Ja	4	3,3
14/w/50	A	Nein (1 mm)	Nein	Nein	Ja	1	4,0

*Ø VOS* Durchmesser der V. ophthalmica superior, *HN-Paresen* Hirnnervenparesen, *BH* Bindehaut

^a^In Klammern angegeben ist die Differenz in der Untersuchung mit dem Hertel-Exophthalmometer zwischen dem betroffenen und nicht betroffenen Auge

### Weiterführende bildgebende Diagnostik

**Figure 4:**
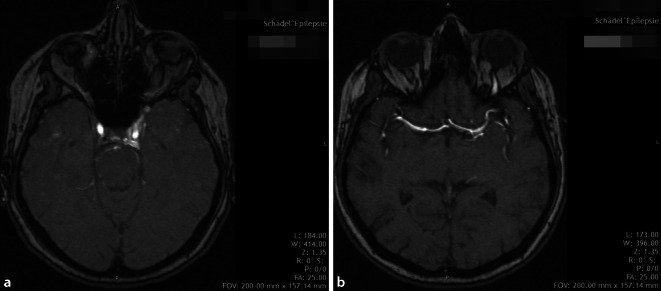

Bei 22 der 25 Patienten (88 %) wurde von den Kollegen der Neurologie die Durchführung einer MR-Angiographie des Halses und der intrazerebralen Gefäße empfohlen (Tab. 6; Abb. 4). Bei Low-flow-Fisteln wurde nur bei einer Patientin aufgrund von Allgemeinerkrankungen auf die MRA verzichtet und initial die DSA indiziert. Bei diskreten Befunden blieb eine Dilatation der V. ophthalmica superior als indirektes Zeichen einer CCF in der nativen bildgebenden Diagnostik gelegentlich unentdeckt; die MR-Angiographie zeigte schließlich eine arterialisierte Füllung des Sinus cavernosus und der venösen Gefäße.

**Table 6:** 

Fisteltyp	CTA	MRA	DSA	Keine Versorgung	Versorgung rein endovaskulär	Chirurgische Freilegung der VOS
A (*n* = 6)	1 (17 %)	4 (67 %)	5 (83 %)	1 (17 %)	4 (67 %)	–
B (*n* = 2)	–	2 (100 %)	2 (100 %)	1 (50 %)	1 (50 %)	1 (50 %)
D (*n* = 17)	–	16 (94 %)	17 (100 %)	1 (6 %)	10 (59 %)	7 (41 %)
Gesamt	1 (4 %)	22 (88 %)	24 (92 %)	3 (12 %)	15 (60 %)	8 (32 %)

*CTA* computertomographische Angiographie, *MRA* Magnetresonanzangiographie, *DSA* digitale Subtraktionsangiographie, *VOS* V. ophthalmica superior

Nachdem auch in der MRA der Verdacht auf eine Carotis-Sinus-cavernosus-Fistel erhärtet werden konnte, erfolgten die Diagnosesicherung und Therapieentscheidung anhand der Untersuchungsergebnisse der DSA. Basierend auf der Klassifikation nach Barrow, wurden die Carotis-Sinus-cavernosus-Fisteln entsprechend der arteriellen Speisung eingeteilt. Typ-D-Fisteln waren am häufigsten vertreten (17 Patienten 68 %), gefolgt von Typ-A- (6 Patienten, 24 %) und Typ-B-Fisteln (2 Patienten, 8 %). Bei keinem der Patienten wurde eine Typ-C-Fistel diagnostiziert.

### Therapie

**Figure 5:**
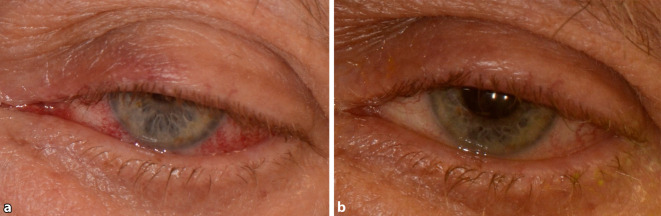

Die Therapieentscheidung richtet sich nach dem Shuntvolumen, dem venösen Drainagemuster und der Gefäßmorphologie. Eine präoperativ durchgeführte Angiographie ist daher erforderlich. In 22 Fällen (88 %) wurde sich für eine operative Versorgung der Fistel entschlossen (Tab. 6). Bei 2 Patienten (8 %) wurde sich aufgrund des hohen Patientenalters, des mangelnden Leidensdrucks und der fehlenden vitalen Bedrohung gegen ein interventionelles Vorgehen ausgesprochen. Ein Patient war nach der diagnostischen DSA nicht mehr in unserer Institution vorstellig. Ein rein endovaskulärer Zugang wurde bei 16 der 22 Patienten gewählt (64 %), eine primäre oder sekundäre chirurgische Freilegung der V. ophthalmica superior war schließlich in 8 Fällen notwendig (36 %). Das Vorschieben des Katheters war bei 2 Patienten nicht möglich, sodass der Fistelverschluss nicht gelang. Bei 20 der 22 operierten Patienten (91 %) konnte der Fistelverschluss erreicht werden (Abb. 5). Aufgrund der fehlenden vitalen Bedrohung wurde beschlossen, zunächst Verlaufskontrollen durchzuführen. Bei 2 Patienten wurden postinterventionelle Komplikationen beschrieben: zum einen eine transiente Schwellung des Sinus cavernosus, zum anderen ein Apoplex a.e. embolischer Genese.

## Diskussion

Das Ziel dieser Untersuchung war es, das Potenzial der Echographie im Rahmen des Untersuchungsablaufs im Hinblick auf Carotis-Sinus-cavernosus-Fisteln zu untersuchen. Anhand der Beobachtungen lässt sich festhalten, dass bei angiographisch gesicherten CCF stets eine dilatierte V. ophthalmica superior echographisch dargestellt werden konnte. In keinem der Fälle zeigte sich ein unauffälliger echographischer Befund.

Von den klassischen Symptomen wurde von allen Patienten mindestens eines angegeben. In der klinischen Untersuchung waren besonders der Exophthalmus bei 18 Patienten, die episklerale Injektion bei 19 und eine Hirnnervenparese bei 22 von 25 Patienten vorhanden. Es fällt auf, dass bei Patienten mit Typ-A-Fisteln okuläre Symptome und/oder ophthalmologische Befunde nicht häufiger vorhanden waren im Vergleich zu Patienten mit indirekten Fisteln. Dies unterstreicht, dass das klinische Bild besonders vom venösen Drainagemuster abhängt, welches in der Klassifikation nach Barrow nicht berücksichtigt wird. Ist das Shuntvolumen gering, können okuläre Symptome mild ausgeprägt sein.

Vor allem in Kombination mit einem Exophthalmus und/oder einer episkleralen Venenstauung sollte an das Vorliegen einer CCF gedacht werden. Bei Low-flow-Fisteln war die Prävalenz an Patienten mit einer arteriellen Hypertonie in der Allgemeinanamnese nicht höher im Vergleich zur Allgemeinbevölkerung [13].

Übereinstimmend mit unseren Beobachtungen wurden Hirnnervenparesen in einer Arbeit von Kurata et al. [14] als häufigster Erstbefund bei Patienten mit indirekten Fisteln beschrieben. In diesem Patientenkollektiv entwickelten sich weitere klassische Symptome erst im Verlauf.

Walker und Allegre [15] beobachteten, dass das Fehlen eines Exophthalmus eine Carotis-Sinus-cavernosus-Fistel unwahrscheinlich macht, Yoshida et al. [16] sahen bei fast allen Patienten eine Bindehautchemosis, und Taniguchi et al. [17] beschrieben die Abwesenheit eines Ohrengeräusches als den Hauptgrund einer initialen Fehldiagnose.

Bei binokularen Doppelbildern kommen differenzialdiagnostisch ein Aneurysma oder Diabetes mellitus, bei einer Bindehautchemosis eine Konjunktivitis und bei einem retroorbitalen/zerebralen Schmerz eine Trigeminusneuralgie oder Migräne in Betracht [14].

Beim Vorliegen einer dilatierten V. ophthalmica superior muss differenzialdiagnostisch an eine orbitale arteriovenöse Malformation, Venenthrombosen oder orbitale Tumoren gedacht werden [18]. Nach auffälligem Befund in der echographischen Untersuchung sollte deshalb zeitnah eine Bildgebung des Schädels folgen. In der Literatur wird eine initiale MR-Angiographie als Screeninguntersuchung (TOF-MRA oder kontrastmittelverstärkte MRA) empfohlen, bevor die invasive DSA mit dem Zweck der Diagnosesicherung und Therapieplanung folgt [9].

### Limitationen und Ausblick

In diese retrospektive Studie wurden nur Patienten mit angiographisch gesicherter Carotis-Sinus-cavernosus-Fistel eingeschlossen. Somit kann keine Aussage über falsch positive Ergebnisse gewonnen werden. Eine Echographie ist bisher in den routinemäßigen Untersuchungsablauf nicht eingebunden, sodass aufgrund des retrospektiven Studiendesigns nicht alle Patienten sonographisch untersucht wurden.

Die Untersuchungsergebnisse verdeutlichen, dass eine echographisch dilatiert erscheinende V. ophthalmica superior zusammen mit den typischen klinischen Befunden wegweisende Informationen zur Diagnosefindung liefern kann. Aufgrund der schnellen Durchführbarkeit und breiten Verfügbarkeit kann schon frühzeitig eine Verdachtsdiagnose gestellt und die notwendige Bildgebung in die Wege geleitet werden. Kann nun eine CCF bei neu aufgetretenen Doppelbildern und einem der „klassischen“ Symptome differentialdiagnostisch in Betracht kommen, muss eine Sonographie der Orbita vor der weitergehenden Diagnostik durchgeführt werden. Bei positivem Befund kann schließlich gezielt die weitere Bildgebung – im Idealfall eine MRA – veranlasst werden. Durch eine zeitnahe Diagnose und Therapieentscheidung kann das Risiko möglicher Folgeschäden gesenkt werden. Ein erhöhter venöser Druck birgt die Gefahr der intrakraniellen Hypertension und intrazerebralen Stauungsblutungen [19]. Ophthalmologische Komplikationen sind ein Sekundärglaukom und eine irreversible Visusminderung.

## Fazit für die Praxis

- Mittels orbitaler Echographie kann die erweiterte V. ophthalmica superior gut dargestellt werden.
- Die Echographie der Orbita muss unverzichtbarer Teil der Diagnostik bei episkleraler Venenstauung, Proptosis und Chemosis sein.
- Die weitere diagnostische Bildgebung (MRA, DSA) wird durch die Echographie nicht ersetzt, kann bei auffälligem Befund aber frühzeitig und gezielt veranlasst werden.
